# Meta-analysis of routine blood tests as predictors of mortality in COPD

**DOI:** 10.3402/ecrj.v1.24110

**Published:** 2014-06-05

**Authors:** Frederikke K. Lomholt, Anne S. Laulund, Nina H. Bjarnason, Henrik L. Jørgensen, Nina Skavlan Godtfredsen

**Affiliations:** 1 Department of Clinical Biochemistry, Bispebjerg University Hospital, Copenhagen, Denmark; 2 Department of Endocrinology and Metabolism, Odense University Hospital, Odense, Denmark; 3 Department of Lung Transplantation, Rigshospitalet, Copenhagen, Denmark; 4 Department of Respiratory Medicine, Hvidovre University Hospital, Copenhagen, Denmark

**Keywords:** COPD, mortality, blood tests, meta-analysis

## Abstract

**Objective:**

The purpose of this study was to examine whether routine blood tests can be useful in predicting mortality in COPD patients.

**Methods:**

Eligible studies were found through a search conducted in the PubMed and Embase databases, the Cochrane Library, and the Web of Knowledge. Twelve studies were included for the meta-analysis of five biochemical markers. Pooled odds ratios (ORs), matching 95% confidence intervals (CIs), and p-values for each of the biochemical markers were calculated using the random effect model.

**Results:**

The following four examined biochemical markers were shown to be associated with mortality in patients suffering from COPD: anemia (OR=2.62, 95% CI: 1.60; 4.29, p=0.01), hypoalbuminemia (OR=2.90, 95% CI: 1.56; 5.40, p=0.0008), elevated NT-proBNP (OR=7.54, 95% CI: 4.04; 14.10, p<0.00001), and elevated cardiac troponin T (OR=3.10, 95% CI: 1.11; 8.25, p=0.03). hs-CRP was not found to be associated with increased mortality.

**Conclusion:**

In this study, we found that anemia, hypoalbuminemia, elevated NT-proBNP, and *elevated* cardiac troponin T were associated with increased mortality in patients suffering from COPD.

Chronic obstructive pulmonary disease (COPD) is characterized by a combination of small airway disease (obstructive bronchiolitis) and parenchymal destruction (emphysema) resulting in a progressive development of airflow limitation. Management of this globally increasing health problem has recently been extensively updated by the Global Initiative for Chronic Obstructive Lung Disease (GOLD) (1). It is well known that COPD is a multi-factorial disease composed of both modifiable and non-modifiable risk factors (2), and, furthermore, existing literature suggests an association between COPD and several comorbidities, such as cardiovascular diseases, cancers, osteoporosis, diabetes, anxiety, and depression (3).

According to WHO estimates, 64 million people were suffering from moderate to severe COPD and in excess of three million people died of COPD in 2005. This number is projected to rise by 30% during the next 10 years (4). In 2030, COPD is estimated to become the fourth leading cause of death and the seventh leading cause of disability adjusted life years (DALYs) worldwide (5).

On an individual level, quality of life is strongly related to disease severity (6, 7), and the risk of acute exacerbation, hospital admittance, and morbidity burden increases with disease severity (8).

The longitudinal ECLIPSE cohort has recently provided new insights into the clinical course of COPD, including the prognosis, impact of comorbidities, and relation with systemic inflammation. It is now recognized that COPD is a heterogeneous disease and that a variety of clinical ‘phenotypes’ exist (9).

Moreover, COPD not only affects the individual but it also represents a substantial socioeconomic burden. A number of studies have shown a strong association between disease severity (classified by GOLD standards) and health costs (10–12).

Several studies (13–22) have suggested that biochemical markers could be associated with all-cause mortality in patients suffering from COPD and since routine blood tests are taken from every patient on admission to a hospital, they could represent an affordable and easily assessable marker of high prognostic value. Anemia is well known to be a risk factor in many diseases (23) and has been shown to be predictive of both in-hospital and long-term mortality as well as of increased risk of comorbidities (13–18). Elevated levels of NT-proBNP, a marker known to be associated with cardiac diseases, has also been shown to be associated with mortality in patients suffering from COPD (19–21).

The purpose of this meta-analysis is to discuss and assess the association of commonly used blood markers with mortality in COPD.

## Methods

A search was conducted in the PubMed and Embase databases as well as the Cochrane library using the following search string:

(copd OR ‘ae copd’ OR ‘chronic obstruct* pulmon* disease’)

AND

(mortality OR death OR fatal* OR survival)

AND

(prognosis OR predict* OR prospect OR expect*)

AND

(marker* OR biochemical* OR anaemia OR anemia OR haemoglobin OR hemoglobin OR albumin OR potassium OR glucose OR CRP OR ‘hs crp’ OR nt-probnp OR IL-6 OR ‘interleukin 6’ OR paO2 OR paCO2)

**Fig. 1:**
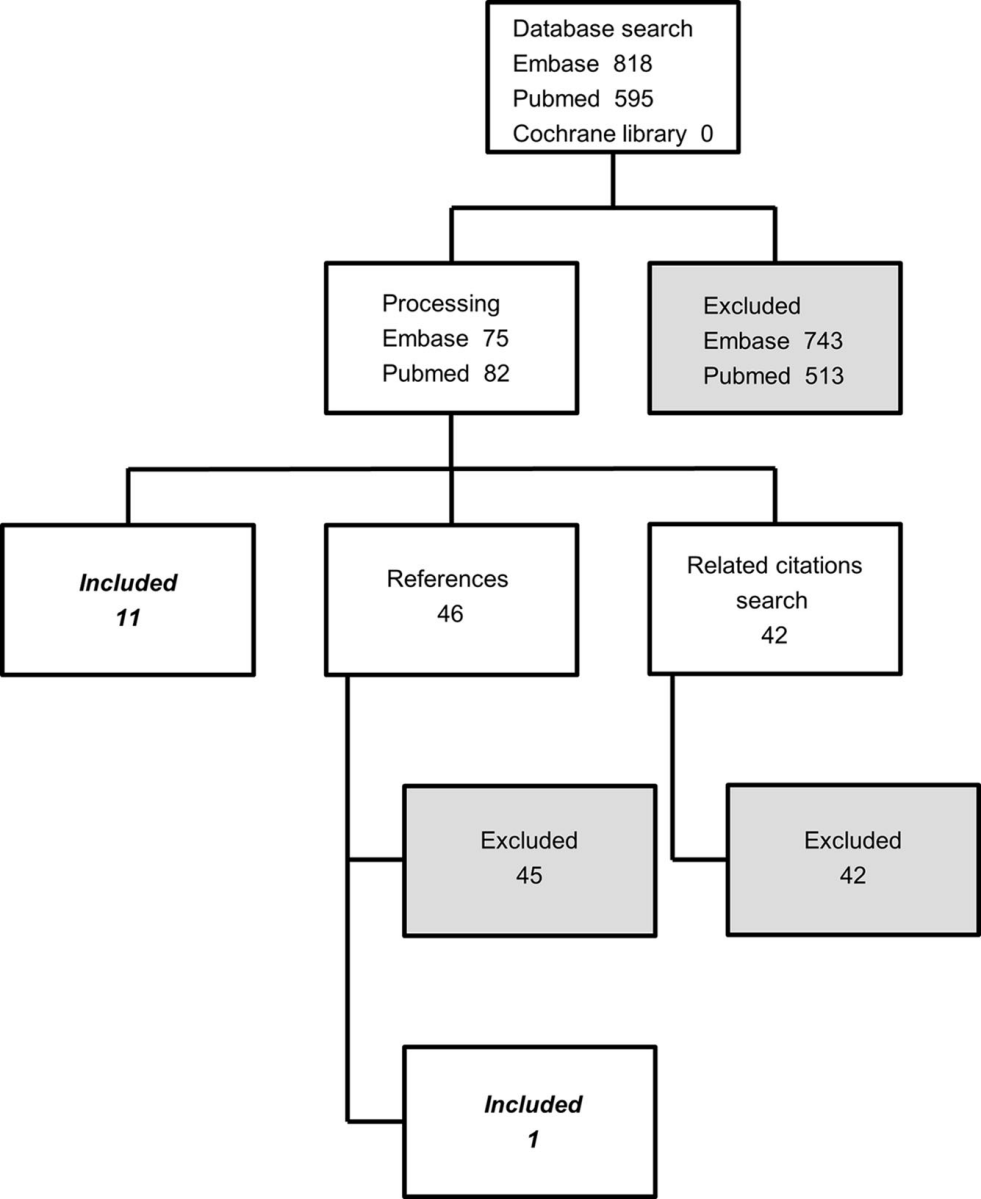
The selection process illustrated as a flow diagram.

 The headlines and abstracts were screened in order to select relevant articles for full-text review. The reference lists of already retrieved articles as well as the ‘related citations’ box in PubMed were screened. In addition, the Web of Science was searched for citing articles of key studies. The search resulted in the inclusion of 12 studies. The selection process is visualized as a flow diagram in Fig. 1.

The inclusion criteria were 1) all patients suffering from COPD, 2) extractable outcome, 3) average age over 65, 4) articles in English, 5) admission values available.

The exclusion criteria were 1) missing data, 2) patients with other respiratory diseases.

The included biochemical markers were:

- Hemoglobin
- Albumin
- hs-CRP
- NT-proBNP
- Cardiac troponin-T

Other biochemical markers such as IL-6, fibrinogen, and TNF-α were considered relevant, but they were excluded either due to an insufficient number of studies or because the studies did not have extractable data.

For the statistical analysis, odds ratios (ORs) with matching 95% confidence interval (CI) were calculated as described by Bland and Altman (24).

The data for each biochemical marker were pooled and *I*^2^-statistics were calculated to evaluate the heterogeneity of the included studies. *I*^2^>50% was defined as a high heterogeneity. Based on heterogeneity, the pooling of data can be performed using either the random effect model or the fixed effect model. The random effect model weights each study equally and incorporates greater variability, which generates wider CIs than the fixed effect model, whereas the fixed effect model weights each study according to size (25). In some of the variables, the included studies showed a high level of heterogeneity and since the variables are very similar the random effect model was chosen in all the analyses. This makes it easier to compare the analyses and furthermore gives the most conservative estimates. For the pooling of data, the unadjusted ORs were used to make the different studies comparable, since the studies with adjusted ORs had adjusted for different factors which made them incomparable.

Funnel plots were used to further check for potential heterogeneity and publication bias. The PRISMA guidelines for meta-analysis were followed (26).

The statistical analyses were calculated using RevMan (The Nordic Cochrane Centre Copenhagen, Denmark) and Excel (Microsoft Redmond, WA, USA).

## Results

### Included studies

Table 1 shows the characteristics of the included studies for each marker with relevant demographics, study design, calculated ORs, and p-values as well as cutoff values.

**Table 1:** Characteristics of the studies included for each of the biochemical markers

Marker	Author	Year	Number of participants (male %)	FEV 1 (% of predicted)	Average age (years)	Length of follow up	OR	*p*	Cutoff males	Cutoff females	Cutoff common
Hemoglobin	Haja et al. (14)	2013	65 (45)	32	71.1	in-hospital	5.54 (1.81; 16.92)	0.003	13 g/dl	11.5 g/dl	
	Martinez et al. (13)	2012	117 (93)	37.2	72	12 months	5.92 (2.31; 15.16)	0.0002			13 g/dl
	Kollert et al. (15)	2012	309 (71.8)	29.9	65.9	min. 5 months	1.14 (0.61; 2.14)	0.7	13 g/dl	12 g/dl	
	Cote et al. (16)	2007	650 (96)	42.3	70.1	37 (± 22) months	1.68 (1.12; 2.52)	0.01			13 g/dl
	Rasmussen et al. (17)	2010	222 (45)	–	66% > 65	90 days	4.00 (1.99; 8.04)	0.0001			12 g/dl
	Boutou et al. (18)	2010	294 (64.6)	36.1	67.9	32.7 months	2.37 (1.22; 4.60)	0.01			13 g/dl
Albumin	Haja et al. (14)	2013	65 (45)	32	71.1	in-hospital	3.98 (1.32; 12.02)	0.01			3.5 g/dl
	Gunen et al. (22)	2005	205 (87.8)	38.2	64.8	6 months	2.57 (1.22; 5.40)	0.01			3.5 g/dl
CRP	Haja et al. (14)	2013	29 (45)	32	71.1	in-hospital	3.43 (0.39; 30.55)	0.3			3 mg/L
	Liu et al. (27)	2011	114 (97.4)	53.2	69.6	max. 36 months	5.53 (1.65; 18.56)	0.006			3 mg/L
	de Torres et al. (28)	2008	218 (64)	46	65	36 (24–50) months	1.17 (0.62; 2.21)	0.6			3 mg/L
	Høiseth et al. (20)	2012	99 (53)	33.3	71.5	22.8 months	0.84 (0.32; 2.17)	0,7			3 mg/L
NT-proBNP	Chang (19)	2011	244 (45)	35	71.7	30 days	6.75 (2.58; 17.61)	0.0001			>220 pmol/L
	Høiseth et al. (20)	2012	99 (53)	33.3	71.5	22.8 months	9.40 (3.68; 23.99)	0.00			>300 pg/ml
	Marcun (21)	2012	127 (70)	34	70	6 months	6.02 (1.31; 27.62)	0.02			sex and age adjusted
c-TNT	Marcun (21)	2012	127 (70)	34	70	6 months	1.06 (0.38; 2.98)	0.9			>12 ng/L
	Chang (19)	2011	244 (45)	35	71.7	30 days	5.85 (2.20; 15.57)	0.0004			>0.03µg/L
	Høiseth et al. (20)	2012	99 (53)	33.3	71.5	22.8 months	4.59 (1.75; 12.02)	0.002			>14 ng/L

The studies included on NT-proBNP and albumin showed a high level of similarity (I^2^=0%), whereas the studies included on anemia, hs-CRP, and cardiac troponin T showed higher levels of dissimilarity (*I*^2^>50%).

### The effect of anemia on mortality (Fig. 2)

The included studies showed high level of heterogeneity (I^2^=68%) and the studies with small study populations had higher ORs than the studies with larger study populations. Cutoff values were either defined as a common cutoff or gender specific cutoff values. ORs were ranging from 1.14 in the study by Kollert et al. (15) to 5.92 in the study by Martinez-Rivera et al. (13). One study (15) did not show a significant correlation between anemia and morality with an OR of 1.14 (0.61; 2.14), p=0.67. The pooled analysis did, however, show a significant correlation between anemia and mortality with p=0.01 and a pooled OR value of 2.62 (1.60; 4.29).

**Fig. 2:**
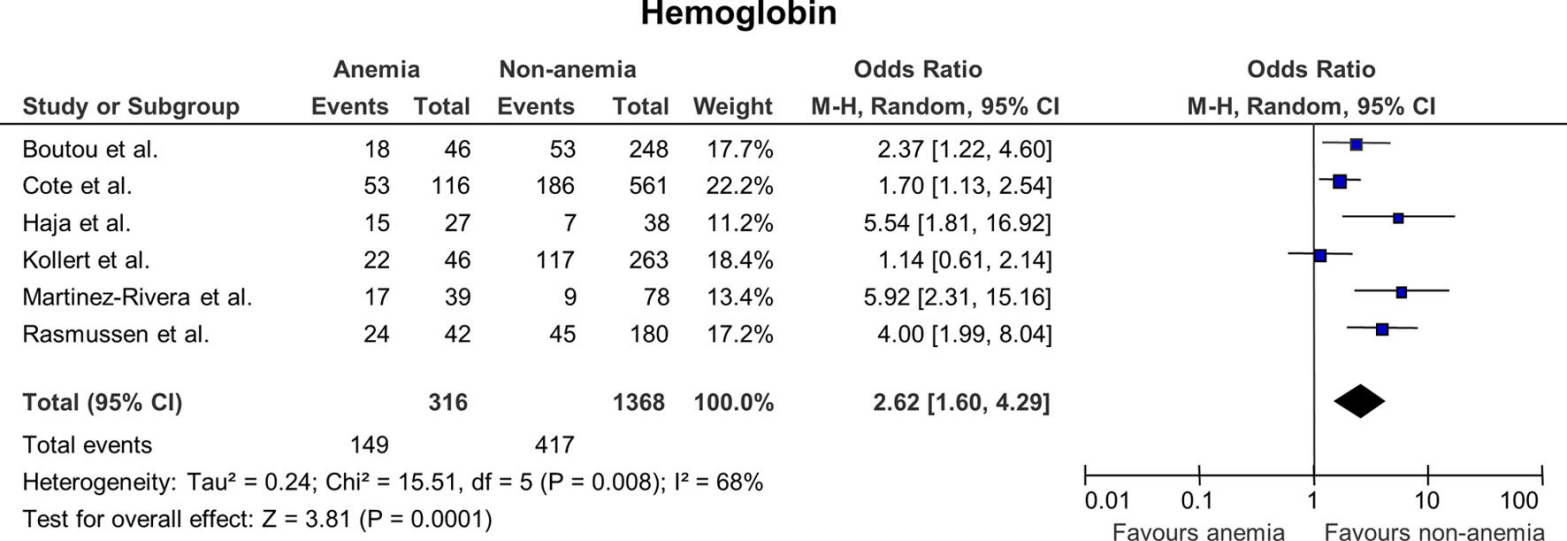
Odds ratios for mortality in patients with anemia versus patients with normal hemoglobin levels.

### The effect of C-reactive protein on mortality (Fig. 3)

Only one study (27) showed a significant correlation between elevated hs-CRP and mortality, whereas the other three studies did not show a significant association with p-values ranging from 0.27 to 0.72. The cutoff value for all included studies was 3 mg/L. The overall analysis had a pooled OR of 1.72 (0.75; 3.95), which was not significant (p=0.20).

**Fig. 3:**
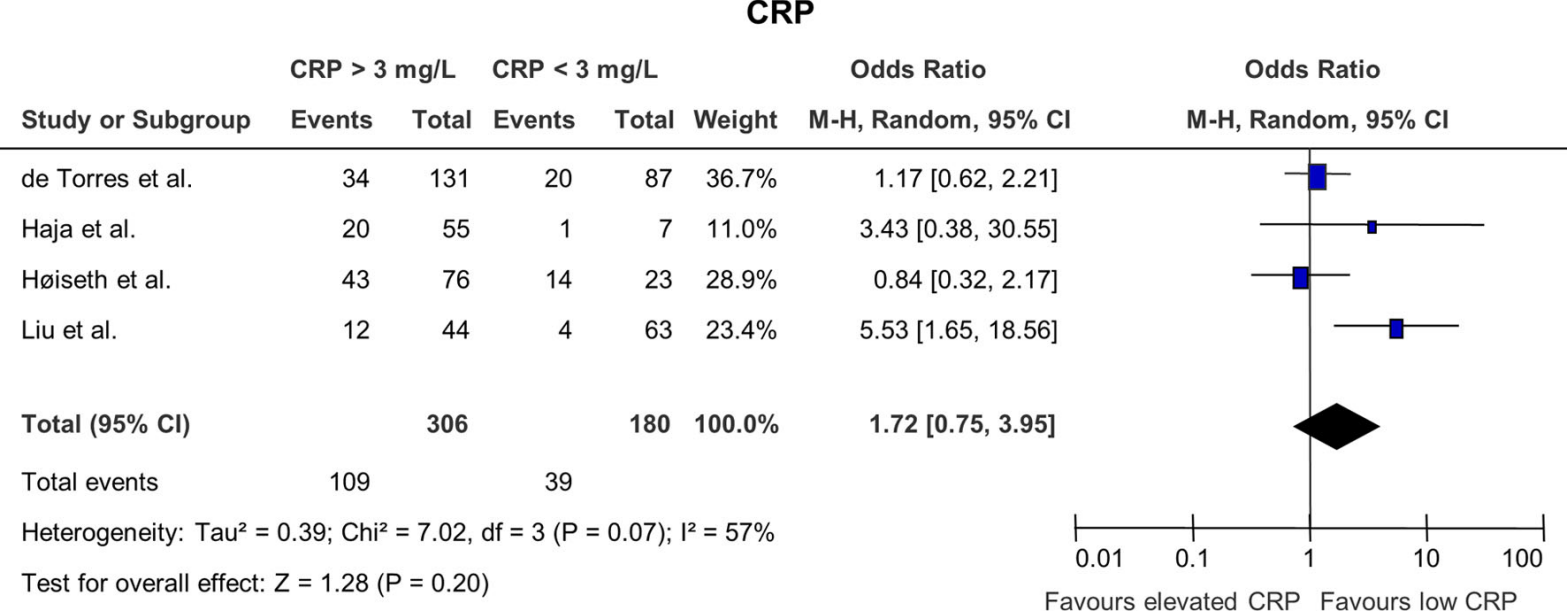
Odds ratios for mortality in patients with elevated CRP versus patients with low CRP. Only one study showed statistically significance.

### The effect of albumin on mortality (Fig. 4)

The studies included in this analysis had a very high level of similarity. However, only two studies had evaluated the value of albumin on mortality. The cutoff value was 3.5 mg/L for both studies. This analysis had the lowest number of study participants with only 279 subjects. The pooled OR was, however, significant with a value of 2.95 (1.59; 5.46) and a p-value of 0.0006.

**Fig. 4:**
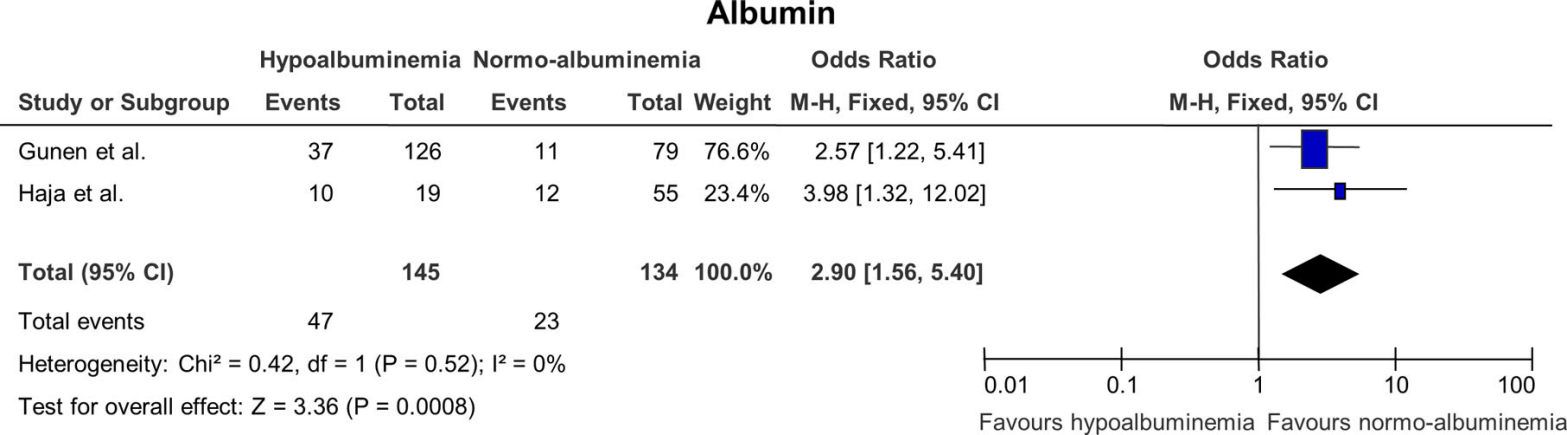
Odds ratios for mortality in patients with hypoalbuminemia versus patients with normal albumin levels.

### The effect of NT-proBNP on mortality (Fig. 5)

Three studies were included in this analysis and they all showed a significant correlation between NT-proBNP and mortality. A total of 43% of the study participants had elevated NT-proBNP indicating that cardiovascular comorbidities are quite common in patients suffering from COPD. For this analysis, the correlation between elevated NT-proBNP and mortality had a pooled OR of 8.59 (4.53; 16.27) with a significant p-value of<0.00001.

**Fig. 5:**
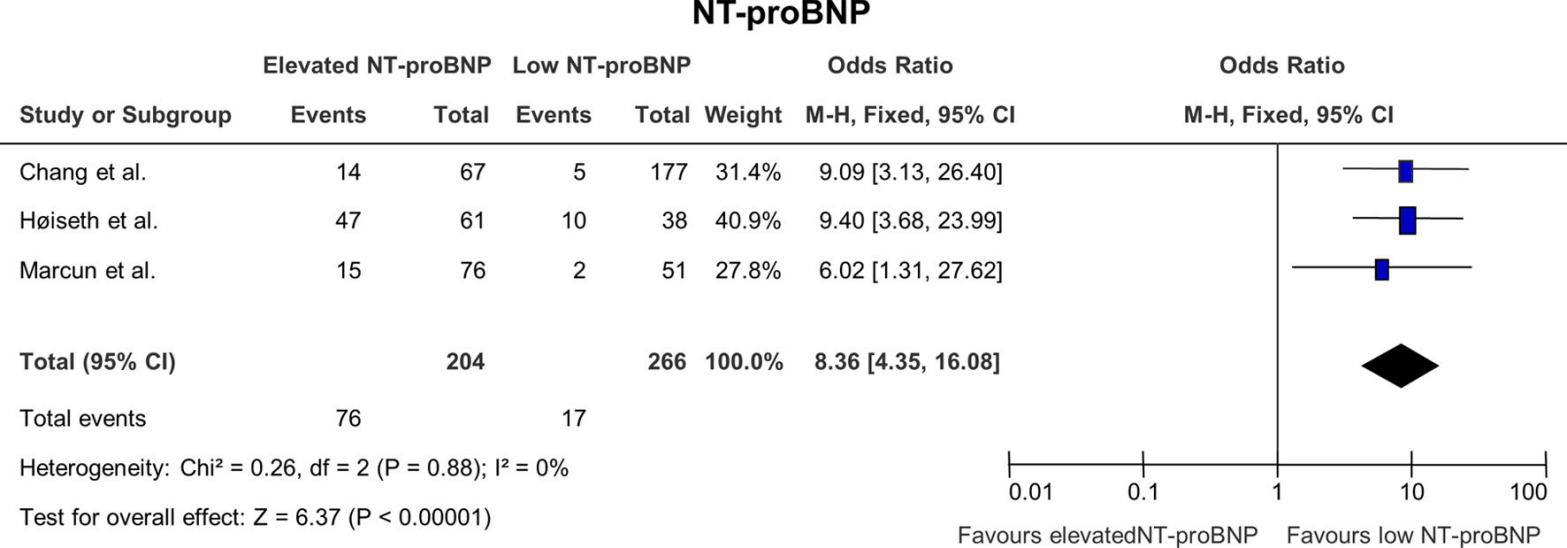
Odds ratios for mortality in patients with elevated NT-proBNP versus patients with low NT-proBNP.

### The effect of cardiac troponin T on mortality (Fig. 6)

Three studies were included in this analysis and two of the studies showed a significant correlation between elevated c-TnT and adverse outcome. Even though one study showed no statistical significance, the overall effect was significant (p=0.03) with a pooled OR value of 3.10 (1.11; 8.25).

**Fig. 6:**
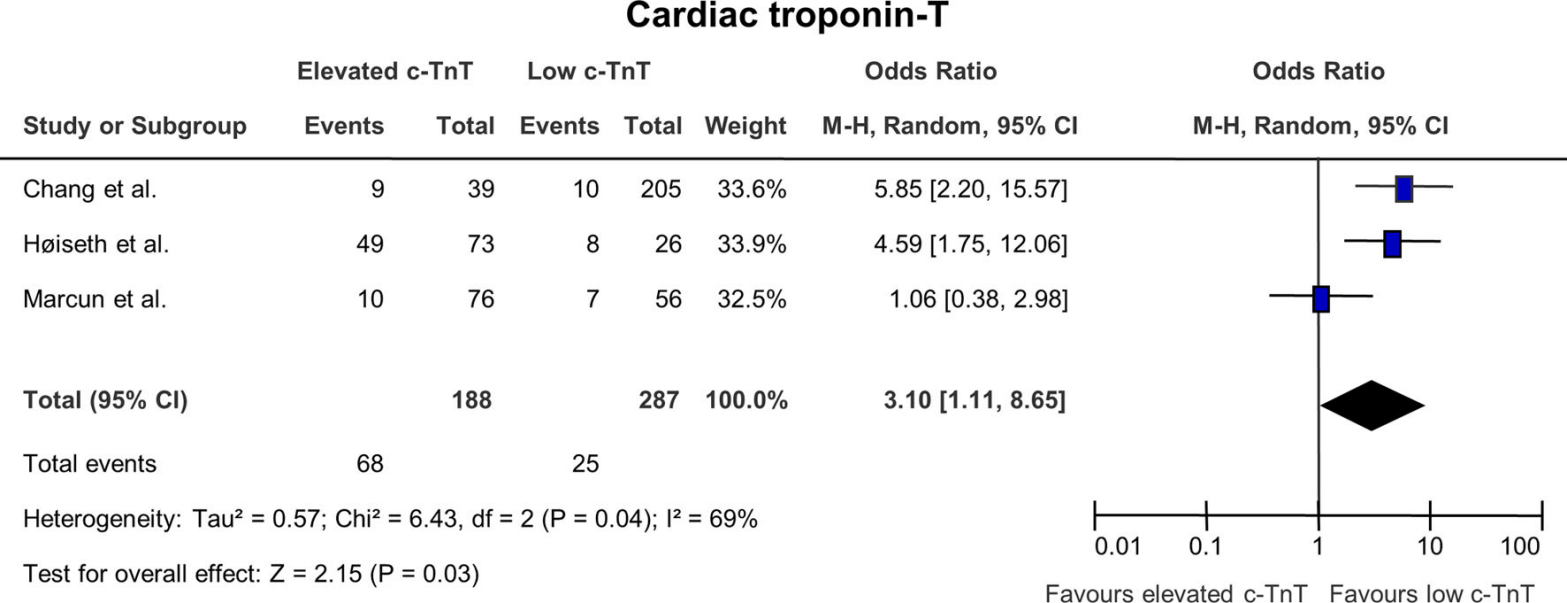
Odds ratios for mortality in patients with elevated cardiac troponin T versus patients with normal c-TnT.

## Discussion

The advantages of using routine blood samples as markers of mortality are many. They are easily interpreted, relatively inexpensive and reproducible compared to other scoring systems. Blood markers might therefore serve as a supplement to the known scoring systems to aid a more precise detection of patients with a high risk of mortality. In this study, high NT-proBNP, hypoalbuminemia, anemia, and high c-TnT all showed a significant correlation with mortality whereas elevated CRP did not.

Hypoalbuminemia and anemia represent reversible conditions where intervention could improve the outcome. Secondary polycythemia is known to occur in patients suffering from severe COPD as a compensatory reaction. However, some patients are anemic and the correlation between anemia and mortality was investigated to show if patients that are not capable of increasing their hemoglobin level incur a greater risk of adverse outcome. Studies evaluating the effects of treating anemia in patients suffering from heart failure indicate beneficial effects on exercise tolerance, symptoms, and clinical outcomes (29, 30). Few studies have addressed whether raising hemoglobin levels in COPD patients show the same beneficial effects (31), but they do show positive benefits of treating anemia in COPD patients (32, 33). However, it is unclear whether anemia is an epiphenomenon or an example of anemia of chronic disease, but the anemic patients did in general present with more severe disease burden, lower levels of FEV_1_, higher age, and more use of LTOT.

Elevation of the cardiac markers NT-proBNP and c-TnT could indicate that many COPD patients are suffering from cardiovascular comorbidities. NT-proBNP is a biochemical marker associated with several diseases but specifically a strong marker of heart failure (34) and elevated levels in COPD patients might indicate undiscovered cardiac disease and the need for further examination of the patients to prevent mortality caused by cardiac comorbidities. It has been shown that cardiac insufficiency relatively often coexists with COPD and that this imposes a greater risk of adverse outcome (35, 36). It is therefore suggested that NT-proBNP and echocardiography should be assessed in all COPD patients.

The three studies included in the analysis of NT-proBNP (20–22) all describe NT-proBNP as an independent marker of mortality. In the study by Chang et al. (19), the authors describe both NT-proBNP and c-TnT as predictors of early mortality independently of any other prognostic factors. Furthermore, none of the included patients were diagnosed with acute coronary syndrome again indicating that these biochemical markers might be able to detect undiscovered coronary diseases. In the study by Høiseth et al. (20), the authors found that c-TnT was more strongly associated with mortality than NT-proBNP and conclude that elevated levels of NT-proBNP are independently associated with mortality after multivariable adjustment. In the same way, the study by Marcun et al. (21) shows a lower but still significant correlation between mortality and elevated levels of NT-proBNP after multivariate adjustment.

In the study by Brekke et al. (37), elevated c-TnT was shown to be associated with anemia, tachycardia, renal dysfunction and myocardial injury. Furthermore, c-TnT was associated with elevated neutrophil blood count indicating a systemic inflammatory response that was not caused by infection. The study by Sin et al. (3) also suggests the correlation between systemic inflammation and cardiovascular diseases and anti-inflammatory treatment might thus be a possible approach to improve outcome.

In the study by Gan et al. (38), hs-CRP was shown to be elevated in COPD patients compared to healthy controls. Since systemic inflammation is a suspected risk factor in COPD, the value of hs-CRP as a prognostic marker was investigated. The hypothesis was that COPD patients suffering from infection are at a higher risk of adverse outcome than COPD patients not suffering from infection. The correlation between elevated hs-CRP and mortality was not shown in this study. The possible explanations could be that 1) there is no association, 2) that the chosen cutoff value in this analysis was too low or 3) that the study participants in the included studies were very heterogeneous. These possible explanations could influence the pooled result.

The main limitations to this meta-analysis are that only a few studies met the inclusion criteria and that the studies included were relatively small. The latter of course presents a risk of both over and under interpreting the calculated ORs since a change in classification of even a small group of patients will have an impact on the OR. The use of small studies additionally presents a risk of publication bias, since small studies are more likely to be published when they show positive results with the risk of over interpretation of the effects of abnormal blood tests. Another important limitation is the fact that confounding factors such as age, lung function, and smoking status might not always be adjusted for in the original studies, and thereby increase the ORs in the meta-analyses. Furthermore, some studies were excluded for not having available data and these factors could of course influence the final results. This meta-analysis is by its nature based on observational studies and not randomized trials, which further involves the risk of bias.

## Conclusion

High NT-proBNP, hypoalbuminemia, anemia, and high c-TnT all showed a significant correlation with mortality. This study therefore suggests that these biochemical markers in the future might have a role in the prediction of mortality in patients suffering from COPD. The patients included in the studies were all suffering from severe to very severe COPD, which means that the conclusions might not be generalizable to milder COPD-cases. Further prospective studies should be performed to evaluate the effect of using blood tests as prognostic tools in both severe and mild COPD as well as taking several potential confounders into account.
